# Differential Expression Profiles and Functional Prediction of Circular RNAs in Pediatric Dilated Cardiomyopathy

**DOI:** 10.3389/fmolb.2020.600170

**Published:** 2020-12-18

**Authors:** Wei Sun, Bo Han, Dongxiao Cai, Jing Wang, Diandong Jiang, Hailin Jia

**Affiliations:** Department of Pediatric Cardiology, Shandong Provincial Hospital, Cheeloo College of Medicine, Shandong University, Jinan, China

**Keywords:** biomarkers, microarray, pediatric dilated cardiomyopathy, circular RNAs (circRNAs), gene expression profile (GEP)

## Abstract

Circular RNAs (circRNAs) have emerged as essential regulators and biomarkers in various diseases. To assess the different expression levels of circRNAs in pediatric dilated cardiomyopathy (PDCM) and explore their biological and mechanistic significance, we used RNA microarrays to identify differentially expressed circRNAs between three children diagnosed with PDCM and three healthy age-matched volunteers. The biological function of circRNAs was assessed with a circRNA–microRNA (miRNA)–mRNA interaction network constructed from Gene Ontology and the Kyoto Encyclopedia of Genes and Genomes. Differentially expressed circRNAs were validated by quantitative real-time polymerase chain reaction (qRT-PCR) in 25 children with PDCM and 25 healthy volunteers. We identified 257 up-regulated (fold change ≤ 0.5, *P* < 0.05) and 899 down-regulated (fold change ≥2, *P* < 0.05) circRNAs in PDCM patients when compared to healthy volunteers. The qRT-PCR experiments confirmed has_circ_0067735 down-regulation (0.45-fold, *P* < 0.001), has_circ_0070186 up-regulation (2.82-fold, *P* < 0.001), and has_circ_0069972 down-regulation (0.50-fold, *P* < 0.05). A functional analysis of these differentially expressed circRNAs suggests that they are associated with hypertrophy, remodeling, fibrosis, and autoimmunity. CircRNAs have been implicated in PDCM through largely unknown mechanisms. Here we report differentially expressed circRNAs in PDCM patients that may illuminate the mechanistic roles in the etiology of PDCM that could serve as non-invasive diagnostic biomarkers.

## Introduction

Pediatric dilated cardiomyopathy (PDCM) is characterized by left ventricular dilation and systolic dysfunction and commonly results in progressive congestive heart failure, arrhythmia, and sudden cardiac death (Towbin et al., 2006; Weintraub et al., 2017; You et al., 2019). PDCM often has a poor prognosis and is the leading cause of cardiac transplantation worldwide (Everly, 2008; Hertz et al., 2009). It is believed that PDCM pathogenesis is caused by a combination of genetic susceptibility and environmental insults (Japp et al., 2016). However, the specific pathogenic mechanisms of the disease remain unclear. PDCM in children appears to have a wider spectrum of causes than dilated cardiomyopathy (DCM) in adults (Griffin et al., 1988; Towbin, 1999), including idiopathic, genetic mutations, myocarditis, neuromuscular disorders, and inborn metabolic dysfunction. Recently, gene modification and epigenetic regulation have become major sources of investigation to identify the mechanism of pathogenesis in PDCM (Hershberger et al., 2013; Wu et al., 2015).

Circular RNAs (circRNAs) are a class of endogenous coding and non-coding RNA created from precursor mRNA back-splicing in eukaryotes (Chen, 2016). Structurally, circRNAs form unique covalent rings without 5′ caps or 3′ polyadenylated tails (Memczak et al., 2013) and thus exhibit greater stability and sequence conservation than normal linear RNA molecules (Guo et al., 2014). Moreover, circRNAs are generally cell and tissue specific (Wilusz and Sharp, 2013). CircRNAs are involved in numerous regulatory processes, including transcriptional modulation, splicing interference, miRNA sequestration, and translation (Fang, 2018; Li et al., 2018). In recent years, a growing number of studies demonstrate that some circRNAs may act as regulatory “miRNA sponges” that naturally sequester and competitively suppress miRNA activity, suggesting that circRNAs might play important roles in post-transcriptional regulation (Memczak et al., 2013). For example, Zheng et al. reported that circHIPK3 directly binds to miR-124 and inhibits miR-124 activity, modulating cell growth (Zheng et al., 2016). Additionally, ciRS-7 was reported by Hansen TB *et al*. to strongly suppress miR-7 activity by acting as a sponge, resulting in increased levels of miR-7 targets (Hansen et al., 2013). CircRNAs have also been reported to regulate gene expression by interacting with RNA binding proteins and translational regulators or by binding directly to mRNAs (Beltran-Garcia et al., 2020; Zang et al., 2020). Moreover, circRNAs can be translated *in vitro* and *in vivo* (Pamudurti et al., 2017).

Recent research on circRNAs has advanced our understanding of the mechanistic roles they play in cardiovascular diseases (Devaux et al., 2017; Aufiero et al., 2019; Zhang et al., 2019). For example, the circRNA Foxo3 was found to effectively reduce doxorubicin-induced cardiomyopathies, and it plays an important role in the senescence of mouse embryonic fibroblasts (Du et al., 2017). Additionally, Wang K recently verified that heart-related circRNA could protect the heart from pathological hypertrophy and heart failure by inhibiting miR-223 activity (Wang et al., 2016). However, in-depth investigations into the role circRNAs play in PDCM pathogenesis are needed to develop early diagnostic techniques and advance new therapeutic targets.

Here we assess the expression patterns and functions of circRNAs in PDCM patients and compare them to healthy control participants using circRNA microarray analysis. We constructed a circRNA–microRNA (miRNA)–mRNA interaction network with Gene Ontology (GO) and the Kyoto Encyclopedia of Genes and Genomes (KEGG). Finally, we verified four differentially expressed DCM-associated circRNAs in blood samples from 25 PDCM patients and 25 healthy volunteers by quantitative real-time polymerase chain reaction (qRT-PCR). Our findings provide a framework for the role circRNAs may play in PDCM pathogenesis. Furthermore, we identified three potential biomarkers of pediatric DCM in the peripheral blood that may serve future diagnostic significance.

## Materials and Methods

### Patients and Peripheral Blood Samples

Twenty-five peripheral blood samples were obtained from children with PDCM and 25 from healthy children between March 2019 and June 2020. All PDCM cases were clinically diagnosed in strict accordance with the World Health Organization guidelines (Richardson et al., 1996). The inclusion criteria included the following: (i) younger than 18 years, (ii) left ventricular end-diastolic diameter z-score >+2, after body surface area correction (Everitt et al., 2014), and (iii) left ventricular ejection fraction ≤ 45%. Patients with hypertension, congenital heart disease, ischemic heart disease, and malformations were excluded. The healthy volunteers were age- and sex-matched with the PDCM cases. The clinical characteristics of the 25 patients and the 25 volunteers are summarized in Table 1. Six samples were subjected to microarray analysis, and all 50 samples were used for subsequent RT-qPCR validation. This study was approved by the Institutional Ethics Committee (NSFC: NO. 2018-115), and the participants provided informed consent or assent (parental informed consent for minors).

**Table 1:** Clinical characteristics of the pediatric dilated cardiomyopathy (PDCM) and control groups.

	**PDCM group (*n* = 25)**	**Control group (*n* = 25)**	***P*-value**
**Sex**			
Male	10	11	
Female	15	14	
Age (years) (median, *P*_25_, *P*_75_)	1.42 (0.71, 7.42)	1.5 (0.63, 7.67)	
**Echo**			
LVEDD Z-score (mean ± SD)	6.74 ± 2.69	−0.50 ± 0.84	***
LVEF (%) (mean ± SD)	29.4 ± 7.88	63.92 ± 0.64	***
NT-ProBNP (pg/ml) (mean ± SD)	12,003 ± 11,373	50.7 ± 37.02	***

*Echo, echocardiographic; LVEDD, left ventricular end-diastolic diameter; Z-score, after body surface area correction, the distance from the average (normal range, 0 ± 2); LVEF, left ventricular ejection fraction (normal value >60%); NT-ProBNP, brain natriuretic peptide, an index of heart failure (normal range, 0–450 pg/ml)*.

*** *p < 0.001 vs. control*.

### RNA Extraction and Quality Control

Leukocytes were isolated from whole peripheral blood *via* centrifugation (1,500 g for 15 min at 4°C) after lysing the red blood cells with Red Blood Lysis Buffer (Solarbio, China). RNA from the three paired samples was extracted with RNeasy Mini Kit (Qiagen, Germany) per the manufacturer's instructions. RNA from the other 44 samples was extracted using Sparkzol Reagent (SparkJade Science Co., Ltd., China), chloroform, and isopropanol precipitation. The RNA Integrity Number (RIN) of the three paired samples was determined using an Agilent 2100 Bioanalyzer (Agilent Technologies, CA, USA); all samples had RIN ≥7.5. The integrity of all 50 RNA samples was determined by agarose gel electrophoresis; RNA concentration and purity were quantified with a Nanodrop 2000 spectrophotometer (Thermo Scientific, Waltham, MA). The RNA samples were stored at −80°C until further use.

### CircRNA Microarray Analysis

CircRNA microarray analysis was performed at Shanghai Sinomics Corporation (Shanghai, China), using Sino human ceRNA array V3.0 (Sinomics Corporation, China). cRNA synthesis and labeling, chip hybridization, washing, and image scanning were performed per manufacturer's instructions. Microarray data were extracted and visualized using the Feature Extraction software 10.7(Agilent Technologies), and the resulting raw data were subjected to quantile normalization using the limma package in R. Data analysis was performed according to Agilent Technologies at Sinotech Genomics Corporation. Differentially expressed circRNAs were identified using a fold change cutoff of 2 and a *P*-value of 0.05.

### Functional Analysis of Differentially Expressed circRNAs

To determine the functional roles of differentially expressed circRNAs in PDCM, we predicted their respective miRNA response elements (MREs) and built a circRNA–miRNA–mRNA network with Arraystar's proprietary miRNA target prediction software. GO and KEGG pathway enrichment analyses were performed using the R package clusterProfiler.

### Quantitative Real-Time Polymerase Chain Reaction

qRT-PCR was performed to quantify the circRNA expression levels on a LightCycler480 system (Roche Diagnostics, Switzerland) using SYBR Green Pro Taq HS Premix (AG11701, Accurate Biotechnology, Hunan, China). Briefly, 1,000 ng of total RNA was reverse-transcribed into cDNA with random primers. All reactions were performed in triplicate containing 2× SYBR Green Premix, 20 ng of template cDNA, and 8,000 nM primers in a final volume of 20 μl. Reaction mixes were analyzed in a 96-well optical reaction plate. Melting curves were analyzed, and PCR products were validated by Sanger sequencing. All primers for qRT-PCR were synthesized by BioSune Biotechnology Co., Ltd. (China) and are listed in Table 2. In addition, circRNA expression levels were normalized to the housekeeping gene β-actin and determined by the 2^−ΔΔCT^ method.

**Table 2:** Specific primers for qRT-PCR.

**Primer name**	**Forward primer sequence (5^**′**^-3^**′**^)**	**Reverse primer sequence (5^**′**^-3^**′**^)**
homoβ-actin	AGTTGCGTTACACCCTTTCTTG	CACCTTCACCGTTCCAGTTTT
hsa_circ_0067735	CAACGTGGCTGATCAAGATG	GCTGGCTGATTATTGAAGCCT
hsa_circ_0070186	GGGCATTTTGAAGACTTACTG	ATGGATTTCTTTAGCTGCTTT
hsa_circ_0069972	TCTAAACTTCATGGAAACAAGGAAA	TCTCTGCTGAAGACTGGGAA
hsa_circ_0005495	TGTGGCCAGAAATTTGCCAG	AATGGGGTCCGGAACAAAGC

### Statistical Analysis

All statistical analyses were conducted using SPSS software (version 24.0, SPSS, IL, USA) and GraphPad Prism version 8.0 (GraphPad Software, CA, USA). Normal distribution of data was presented as mean ± standard deviation (mean ± SD) and compared by Student's *t*-test (two-tailed, unpaired, equal variance). *P* < 0.05 was considered statistically significant.

## Results

### Expression Profiles of circRNAs in Pediatric Dilated Cardiomyopathy

**Figure 1:**
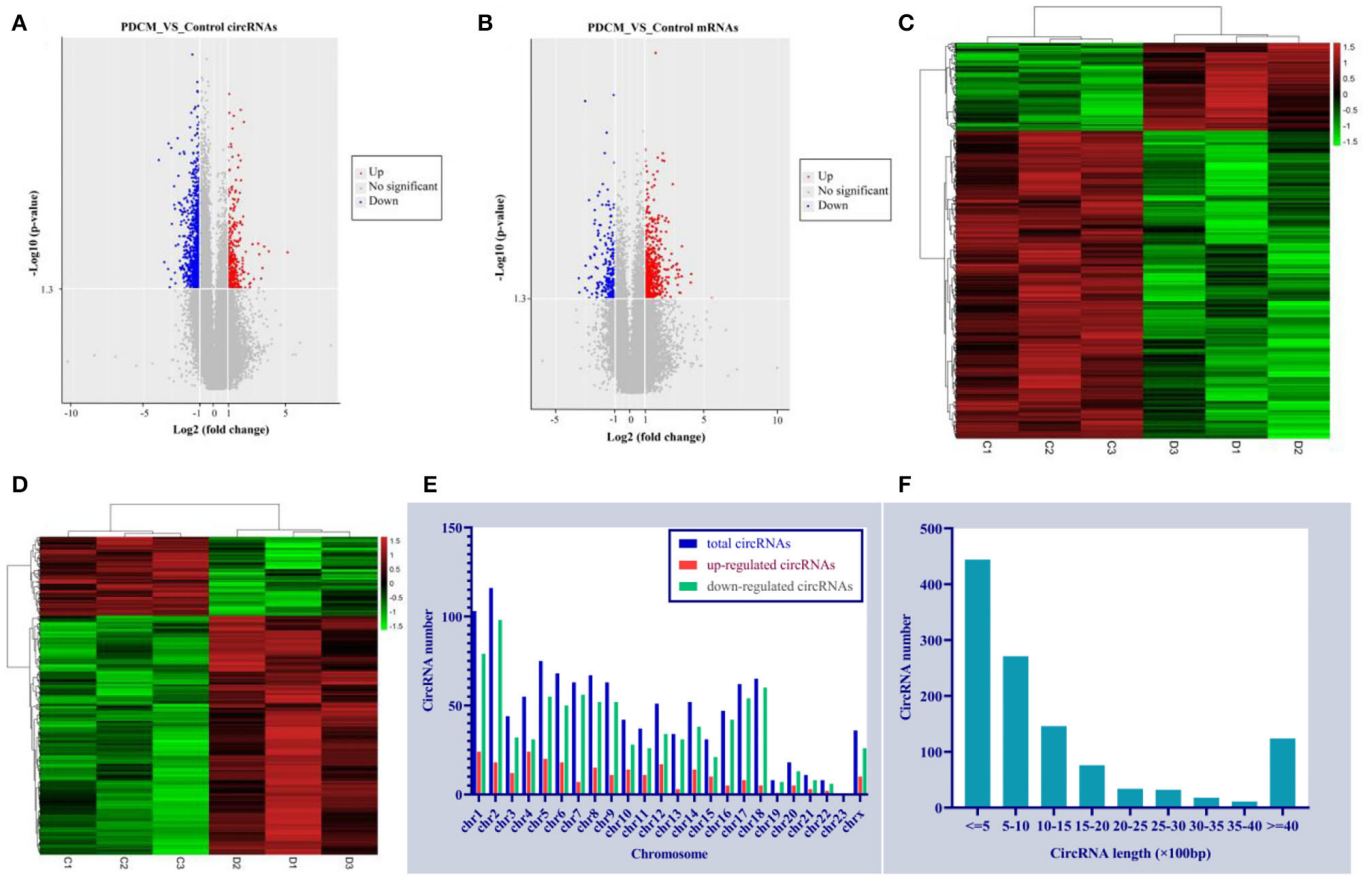
Circulating expression levels of circRNAs and mRNA in the pediatric dilated cardiomyopathy group and control group. **(A)** A volcano plot of differential circRNA expression with various *p*-values and fold changes. **(B)** A volcano plot of differential mRNA expression with various *p*-values and fold changes. **(C)** Hierarchical clustering analysis of circRNAs with altered expression (*p* < 0.05, fold change >2) between the two groups. Red strip, high relative expression; green strip, low relative expression; black strip, no change. Color brightness reflects the degree of expression increase or decrease. **(D)** Hierarchical clustering analysis of mRNAs with altered expression (*p* < 0.05, fold change >2) between the two groups. Red strip, high-relative expression; green strip, low-relative expression; black strip, no-change. Color brightness reflects the degree of expression increase or decrease. **(E)** The dysregulated circRNA distribution rule in human chromosomes. **(F)** Length distribution of the dysregulated circRNAs.

We identified 53,635 circRNAs in three children with PDCM and three healthy volunteers with circRNA microarray analysis. We observed differential circRNA expression levels with various *p*-values and fold changes between the PDCM case and control groups (Figure 1A), in addition to differential mRNA expression (Figure 1B). Hierarchical clustering analysis indicated a distinct circRNA (Figure 1C) and mRNA (Figure 1D) expression profile in PDCM patients when compared to healthy volunteers.

A total of 1,156 differentially expressed circRNAs were detected in PDCM cases compared with control patients (fold change >2, *p* < 0.05), including 257 up-regulated and 899 down-regulated circRNAs (Supplementary File 1). The 10 most up-regulated and 10 most down-regulated circRNAs according to fold change are presented in Table 3. Additionally, 482 mRNAs were up-regulated and 159 were down-regulated in the PDCM group compared to the control group (Supplementary File 2). To explore the molecular characteristics of circRNAs, we assessed the length and the chromosomal distribution of the differentially expressed circRNAs. We found that the differentially expressed circRNAs in PDCM patients were derived mainly from chromosome 2 (10.03%; 116/1,156), chromosome 1 (8.91%; 103/1,156), and chromosome 5 (6.49%; 75/1,156) in descending order (Figure 1E). Additionally, the lengths of these circRNAs were often <2,000 nucleotides (81.06%; 937/1,156), as shown in Figure 1F.

**Table 3:** Serial numbers, *p*-values, fold changes, chromosomes of origin, sequence lengths, and host genes of the top 10 up-regulated and down-regulated circular RNAs associated with pediatric dilated cardiomyopathy.

**CircRNA**	***p*-values**	**FDR**	**Fold change**	**Regulation**	**Chromosome**	**Sequence length**	**Host gene**
hsa_circ_0033063	0.01686	0.44991	38.38073	Up	chr14	587	IFI27
hsa_circ_0088174	0.01637	0.45149	15.19901	Up	chr9	471	ORM1
hsa_circ_0088177	0.01418	0.44940	12.08522	Up	chr9	292	ORM2
hsa_circ_0070185	0.02702	0.43012	11.05281	Up	chr4	531	ANXA3
hsa_circ_0070186	0.01789	0.44561	9.46793	Up	chr4	591	ANXA3
hsa_circ_0070187	0.01300	0.45041	8.94111	Up	chr4	396	ANXA3
hsa_circ_0070170	0.01325	0.44894	6.76701	Up	chr4	1,364	FRAS1
hsa_circ_0025518	0.01989	0.43710	4.148068	Up	chr12	927	PLBD1
hsa_circ_0074239	0.02866	0.43501	4.10679	Up	chr5	692	C5orf32
hsa_circ_0078682	0.02615	0.43219	4.08332	Up	chr6	641	MLLT4
hsa_circ_0058021	0.00107	0.46791	0.06619	Down	chr2	348	CPS1
hsa_circ_0024749	0.00065	0.53912	0.10711	Down	chr11	433	FEZ1
hsa_circ_0069972	0.04774	0.44405	0.11357	Down	chr4	2,014	CXCL5
hsa_circ_0052078	0.03754	0.43460	0.12151	Down	chr19	2,045	PPP2R1A
hsa_circ_0027275	0.00724	0.45933	0.12608	Down	chr12	1,520	MBD6
hsa_circ_0046702	0.00409	0.44719	0.14144	Down	chr18	279	YES1
hsa_circ_0058810	0.03100	0.43439	0.14659	Down	chr2	169	AGAP1
hsa_circ_0067735	0.04257	0.43888	0.15006	Down	chr3	457	MED12L
hsa_circ_0002515	0.02177	0.43474	0.18675	Down	chr4	336	AFAP1
hsa_circ_0030584	0.00476	0.44922	0.19012	Down	chr13	2,004	ABCC4

### Construction of a Functional circRNA–miRNA–mRNA Interaction Network

**Figure 2:**
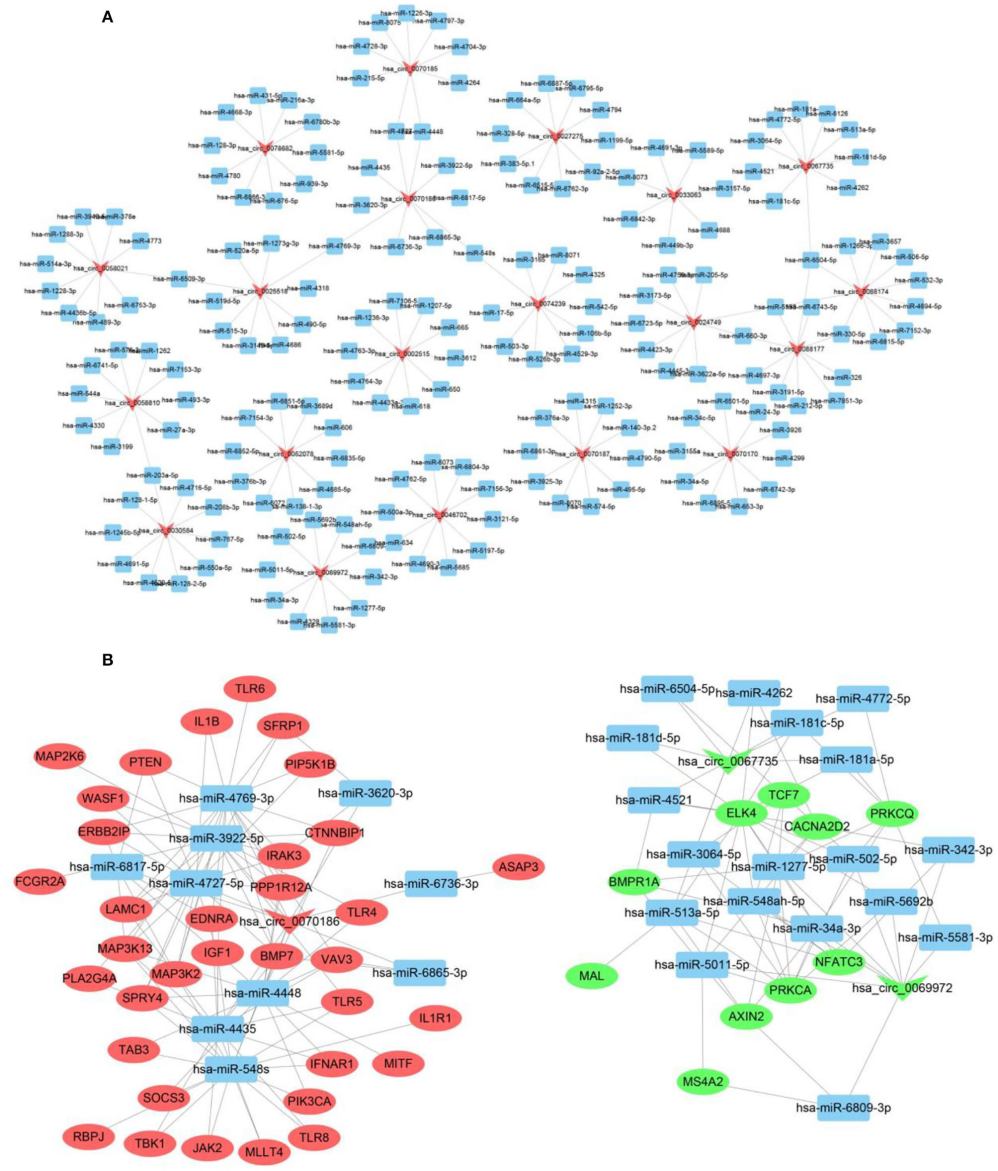
CircRNA–miRNA–mRNA interaction network. **(A)** CircRNA–miRNA interaction network of the top 10 up-regulated and down-regulated circRNAs. **(B)** CircRNA–miRNA–mRNA interaction network of three selected circRNAs. Inverted triangles represent circRNAs; rectangles represent the predicted miRNAs; ellipse nodes represent mRNAs (red, up-regulated; green, down-regulated).

According to previous studies (Hansen et al., 2013), circRNAs can bind to the MREs on miRNAs in a competitive manner to terminate their regulatory effects on target genes. We assessed the likely interactions of differentially expressed PDCM circRNAs with complementary miRNAs using Cytoscape 3.5 and present the five most likely miRNA binding sites for each circRNA in Supplementary File 3. We then constructed our functional network of the top 10 up-regulated and top 10 down-regulated circRNAs with their respective miRNA targets (Figure 2A). We validated our circRNA–miRNA–mRNA interaction network of three differentially expressed circRNAs in 50 samples by qRT-PCR (Figure 2B). Interestingly, the differentially expressed mRNAs that we identified, including CACNA2D2, IGF1, PRKCA, PIK3CA, VAV3, PRKCQ, TLR4, IL1B, TLR8, and CTNNBIP1, are involved in pathways relevant to DCM pathogenesis such as “dilated cardiomyopathy,” “leukocyte transendothelial migration,” “T cell receptor signaling pathways,” “Toll-like receptor signaling pathways,” and “WNT signaling pathways.”

### Validation of circRNA Expression

**Figure 3:**
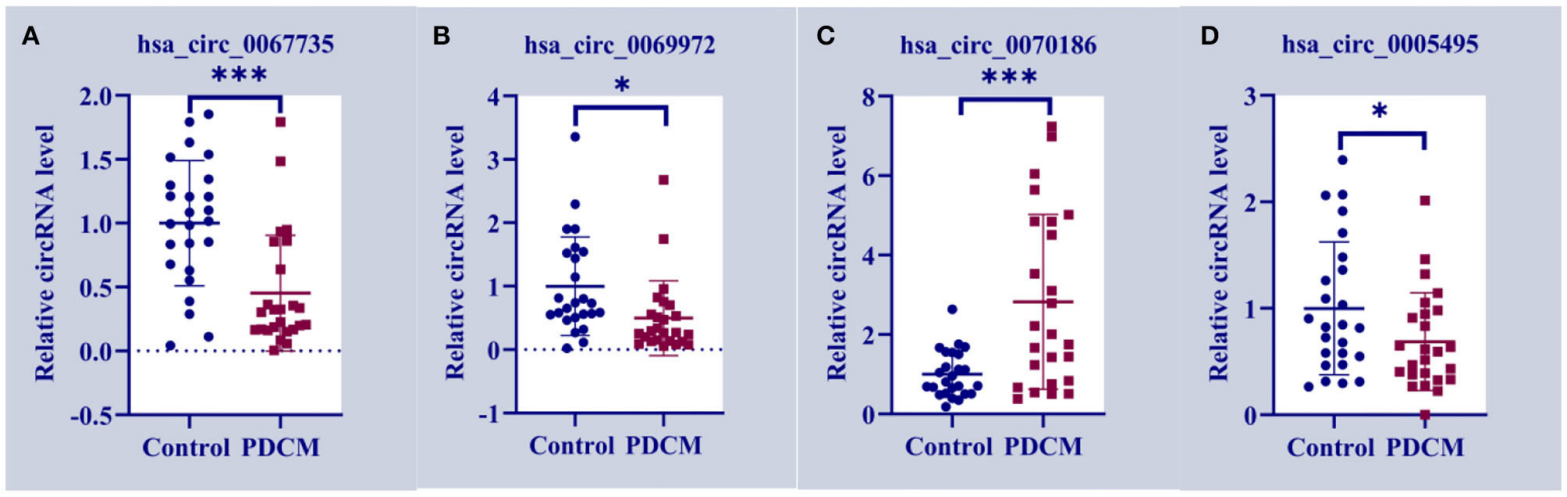
Circulating expression levels of the four circRNAs assessed by qRT-PCR. **(A)** Circulating expression levels of hsa_circ_0067735 in control subjects and the children with pediatric dilated cardiomyopathy (PDCM). **(B)** Circulating expression levels of hsa_circ_0069972 in control subjects and the children with PDCM. **(C)** Circulating expression levels of hsa_circ_0070186 in control subjects and the children with PDCM. **(D)** Circulating expression levels of hsa_circ_0005495 in control subjects and the children with PDCM. Control group, *n* = 25; PDCM group, *n* = 25. *P < 0.05 vs. control; ***p < 0.001 vs. control.

We designed primer pairs for the top 10 differentially up-regulated and down-regulated circRNAs in PDCM. Three pairs successfully amplified the target circRNA sequences, which were validated by RT-qPCR. Two circRNAs of interest, has_circ_0067735 (Figure 3A) and has_circ_0069972 (Figure 3B), were down-regulated 0.45-fold (*p* < 0.001) and 0.50-fold (*p* < 0.05) in the PDCM group, respectively, when compared to healthy participants. Another circRNA, named has_circ_0070186 (Figure 3C), was up-regulated 2.82-fold (*p* < 0.001) in PDCM patient samples. Meanwhile, has_circ_0005495 (Figure 3D) was confirmed to be down-regulated by 0.69-fold (*p* < 0.05), which was opposite to the microarray data.

### Functional Prediction of Differentially Expressed circRNAs

**Figure 4:**
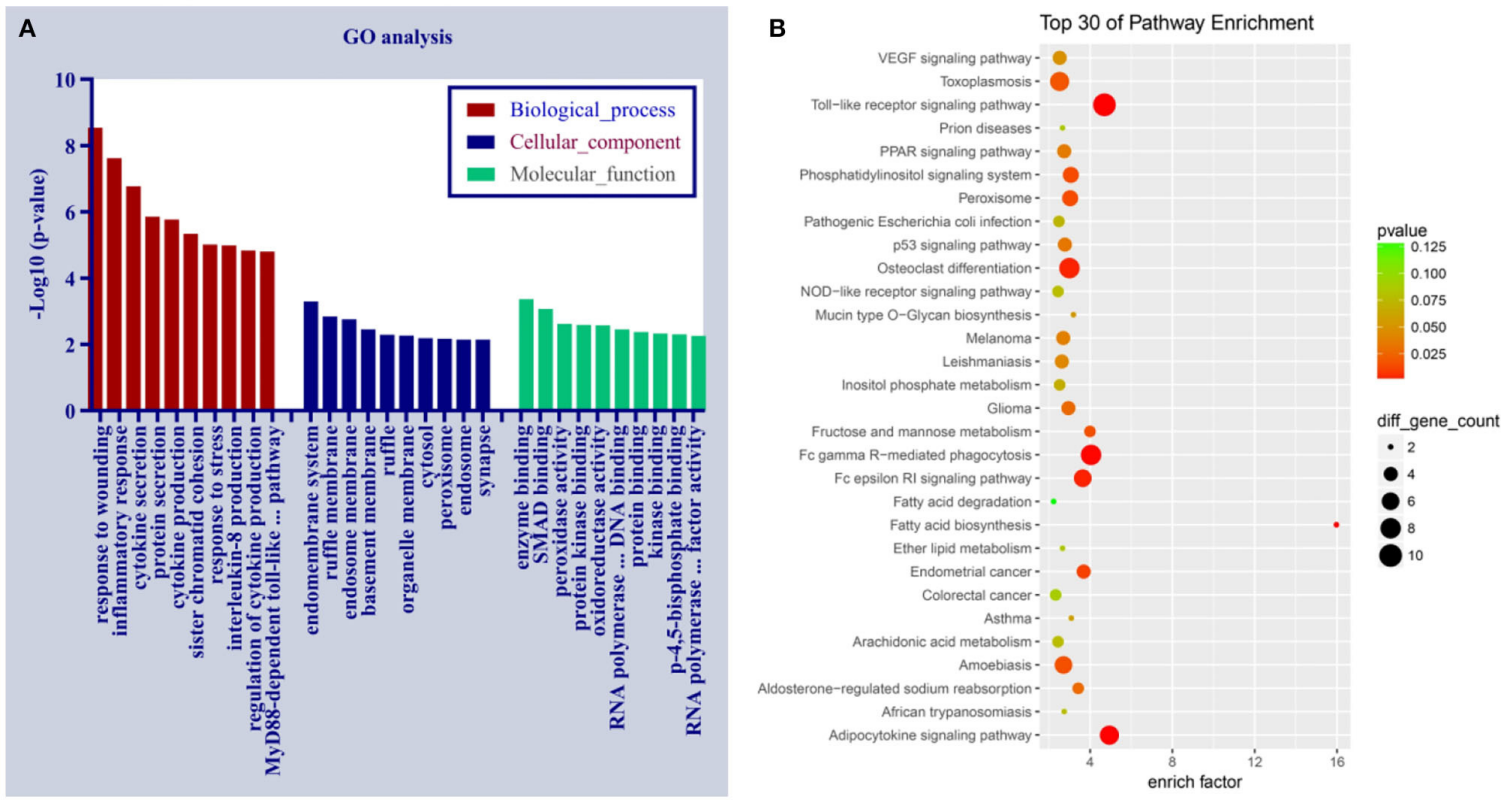
Gene Ontology and the Kyoto Encyclopedia of Genes and Genomes pathway analyses of dysregulated circRNAs in pediatric dilated cardiomyopathy (PDCM). **(A)** Ten most enriched gene ontology terms in biological process, cellular component, and molecular function for differentially expressed circRNAs. **(B)** Top 30 Kyoto Encyclopedia of Gene and Genome pathways of dysregulated circRNAs. EGFR, epidermal growth factor receptor.

To predict the underlying mechanisms of differentially expressed circRNAs in PDCM, we performed a functional annotation analysis of their target mRNAs with GO and the KEGG pathway enrichment tools (Supplementary Files 4, 5). These mRNA targets are likely modulated by circRNAs *via* competitive endogenous RNA (ceRNA) regulation. There are three GO categories, including biological process (BP), cellular component (CC), and molecular function (MF). The top 10 enriched GO terms (in BP, CC, and MF) and the top 30 KEGG pathways of the dysregulated circRNAs in PDCM are displayed in Figure 4. The three most enriched GO terms were “response to wounding,” “inflammatory response,” and “cytokine secretion” in BP; the top three terms in CC were “endomembrane system,” “ruffle membrane,” and “endosome membrane.” In MF, “enzyme binding,” “SMAD binding,” and “peroxidase activity” were the three most enriched GO terms. The KEGG pathways in differentially expressed circRNAs were enriched in “Toll-like receptor signaling” and “Fc gamma R-mediated phagocytosis,” processes commonly associated with PDCM.

## Discussion

It is widely believed that PDCM etiology is driven by both genetic and environmental factors (Poller et al., 2005), with the specific molecular mechanisms largely unknown. Circular RNAs (circRNAs) were recently discovered to be widely expressed across species and have been implicated in several diseases (Pan et al., 2018). However, the expression profiles and functions of circRNAs in PDCM remain elusive. Here we investigated the different expression patterns of circRNAs in peripheral blood leukocytes isolated from PDCM patients when compared to healthy age-matched individuals. A total of 1,156 circRNAs had differential expression in PDCM (fold change >2, *p* < 0.05), including 257 up-regulated and 899 down-regulated transcripts. This is the first study to assess the expression patterns of circRNAs in PDCM and may provide new insights into their mechanistic role in this disease. Four differentially expressed circRNAs from three patients were verified by qRT-PCR in 25 patient samples. Two circRNAs, has_circ_0067735 and has_circ_0069972, were significantly downregulated in DCM, while another, has_circ_0070186, was significantly upregulated. These three circRNAs may serve as potential biomarkers of PDCM in the future. We discovered that these three circRNAs possessed miRNA binding sites, suggesting that they carried a potential for post-transcriptional regulation. has_circ_0067735 likely regulates the expression of CACNA2D2 by binding hsa-miR-4262, while has_circ_0070186 likely regulates IGF1 expression by binding hsa-miR-4448. CACNA2D2 and IGF1 are associated with dilated cardiomyopathy pathway according to KEGG pathway enrichment analyses. However, these associations require further investigation.

The GO biological process and the KEGG pathway enrichment analyses were carried out to explore the potential mechanisms of dysregulated circRNAs in PDCM. PDCM-associated circRNAs were enriched in BP, such as “response to wounding,” “inflammatory response,” and “cytokine secretion.” Meanwhile, the KEGG analysis identified that DCM-associated circRNAs were enriched in pathways such as Toll-like receptor signaling, leukocyte transendothelial migration, T cell receptor signaling, and WNT signaling. These pathways have all previously been implicated in DCM pathogenesis. For example, TLR4 activation causes experimental autoimmune myocarditis progress to DCM in mice (Wu et al., 2018), which was closely related to PDCM. Additionally, WNT signaling is a critical pathway for cardiac hypertrophy and remodeling (Bergmann, 2010; Malekar et al., 2010; Lu et al., 2016). CD4+ T-cells may play a critical role in ADP/ATP carrier-caused mouse DCM (Wang et al., 2006). Inflammatory endothelial activation and migration of immunocompetent cells have been observed in 67% of DCM patients, a process mediated by cell adhesion (Noutsias et al., 1999, 2003). PDCM pathogenesis is usually associated with cardiac hypertrophy, remodeling, fibrosis, and autoimmunity. Therefore, given that the relevant circRNAs we identify relate to these cellular processes, it is likely that their differential expression contributes to PDCM pathogenesis *via* these pathways.

While our findings provide a framework for understanding the role circRNAs play in PDCM etiology, the differential expression of the circRNAs identified here should be verified first in myocardial tissue. Second, these findings should be verified across broader sociodemographic characteristics, including ethnicity. Our participants lacked sociodemographic breadth and were mostly of Asian ethnicity. The expression patterns of the circRNAs we identified here should also be analyzed in other cardiac pathologies to ensure that they are specific to PDCM. Finally, follow-up experiments are needed at the cellular and organismal level to determine the mechanistic functions of these circRNAs in PDCM.

## Conclusion

This study provides the first profile of differentially expressed circRNAs in PDCM. We used GO and KEGG pathway enrichment analyses to construct a circRNA–miRNA–mRNA interaction network to preliminarily assess the roles and potential mechanisms of dysregulated circRNAs in the development of PDCM. We identified three relevant circRNAs in pediatric DCM: has_circ_0067735 and has_circ_0069972 were markedly down-regulated, while has_circ_0070186 was up-regulated, suggesting that they may constitute candidate biomarkers of PDCM. This work provides a foundation for further research on the mechanistic role circRNAs play in PDCM.

No effective circRNA has been utilized for early PDCM diagnosis. However, the circRNAs that we identified have potential as novel non-invasive biomarkers in PDCM.

## Future Perspective

Our study provides a new foundation for understanding the roles circRNAs play in PDCM. We hope to explore their exact mechanism of action in a follow-up series of experiments. We believe that has_circ_0067735, has_circ_0070186, and has_circ_0069972 could serve as novel biomarkers and therapeutic targets in PDCM. Ultimately, understanding the role of differently expressed circRNAs has the potential to improve detection, prevention, and treatment of PDCM early when the maximum clinical benefit can be achieved.

## Data Availability Statement

The raw data supporting the conclusions of this article will be made available by the authors, without undue reservation.

## Ethics Statement

The studies involving human participants were reviewed and approved by the Institutional Ethics Committee, Provincial Hospital affiliated to Shandong First Medical University. Written informed consent to participate in this study was provided by the participants' legal guardian/next of kin.

## Author Contributions

BH and WS conceived and supervised the study. WS and JW designed the experiments. WS and DC performed the experiments. DJ and HJ analyzed and interpreted the data. WS reviewed and edited the manuscript for important intellectual content. BH and JW provided substantive revisions to the manuscript. All the authors approved the final version of the manuscript.

## Conflict of Interest

The authors declare that the research was conducted in the absence of any commercial or financial relationships that could be construed as a potential conflict of interest.

## Supplementary Material

Supplementary File 1 Differentially expressed circRNAs in pediatric dilated cardiomyopathy.

Supplementary File 2 Differentially expressed mRNAs in pediatric dilated cardiomyopathy.

Supplementary File 3 miRNA binding sites for circRNAs.

Supplementary File 4 Gene Ontology analysis.

Supplementary File 5 Kyoto Encyclopedia of Genes and Genomes pathway.
